# The Role of Leptin in Fetal Growth during Pre-Eclampsia

**DOI:** 10.3390/ijms22094569

**Published:** 2021-04-27

**Authors:** Victoria E. de Knegt, Paula L. Hedley, Jørgen K. Kanters, Ida N. Thagaard, Lone Krebs, Michael Christiansen, Ulrik Lausten-Thomsen

**Affiliations:** 1 Department for Congenital Disorders, Danish National Biobank and Biomarkers, Statens Serum Institute, Artillerivej 5, 2300 Copenhagen, Denmark; PHY@ssi.dk (P.L.H.); MIC@ssi.dk (M.C.); 2 Laboratory of Experimental Cardiology, Department of Biomedical Science, University of Copenhagen, Blegdamsvej 3B, 2200 Copenhagen, Denmark; jkanters@sund.ku.dk; 3 Department of Gynecology and Obstetrics, Copenhagen University Hospital Slagelse, Ingemannsvej 18, 4200 Slagelse, Denmark; idanaeslund@gmail.com; 4 Department of Obstetrics and Gynecology, University Hospital Hvidovre, Kettegård Alle 30, 2650 Hvidovre, Denmark; lone.krebs@regionh.dk; 5 Department of Biomedical Sciences, University of Copenhagen, Blegdamsvej 3B, 2200 Copenhagen, Denmark; 6 Department of Neonatology, University Hospital Rigshospitalet, Blegdamsvej 9, 2100 Copenhagen, Denmark; ulrik.lausten-thomsen@regionh.dk

**Keywords:** anthropometry, birth weight, fetal development, fetal growth restriction, infant growth, leptin, pre-eclampsia, prenatal growth

## Abstract

Leptin is secreted by the placenta and has a multi-facetted role in the regulation of functions related to pregnancy. Metabolic disorders and insufficient homeostatic compensatory mechanisms involving leptin during pregnancy play a decisive role in the development of pre-eclampsia (PE) and give rise to compromised intrauterine growth conditions and aberrant birth weight of offspring. This review was compiled to elucidate the metabolic background of PE and its relationship with adverse intrauterine growth conditions through the examination of leptin as well as to describe possible mechanisms linking leptin to fetal growth restriction. This review illustrates that leptin in PE is dysregulated in maternal, fetal, and placental compartments. There is no single set of unifying mechanisms within the spectrum of PE, and regulatory mechanisms involving leptin are specific to each situation. We conclude that dysregulated leptin is involved in fetal growth at many levels through complex interactions with parallel pregnancy systems and probably throughout the entirety of pregnancy.

## 1. Introduction

Normal fetal growth depends on complex interactions between maternal, placental, and fetal environments. Pre-eclampsia (PE) is a manifestation of dysregulation in this normally finely tuned balance. Leptin, a peptide hormone most well-known for its role in energy homoeostasis and secretion from adipose tissue, is also secreted by the placenta [1]. The role of placental leptin in both mother and fetus are yet to be fully elucidated, but it is generally agreed that placental leptin is an important hormone and cytokine with a multi-facetted role in the regulation of functions related to pregnancy [2,3,4,5,6]. Metabolic disorders and insufficient homeostatic compensatory mechanisms involving leptin during pregnancy are thought to play a decisive role in the development of PE [2,3] and give rise to compromised intrauterine growth conditions and aberrant birth weight and body composition of offspring.

In this review, we examine the latest information available about leptin action in PE to support the idea that dysregulated leptin is linked to aberrant intrauterine growth and subsequent low birth weight of offspring. Furthermore, this review highlights areas within the field where results are conflicting or where further investigation is needed in order to unravel the complex and interacting network of pathways linking leptin to fetal growth in pre-eclamptic pregnancies. An introductory overview of PE, fetal growth restriction (FGR), leptin and its receptors, and the role of leptin in normal pregnancy is provided. This is followed by a comprehensive examination of leptin action in PE and its association with FGR under the subsections of maternal, fetal, and placental leptin. Finally, possible mechanisms for dysregulated leptin in PE and implications for fetal growth are discussed.

A three-component search was performed in PubMed using the terms “pre-eclampsia”, “leptin”, and “birth weight” as well as their derivatives. The search produced 128 hits spanning from 1994, when leptin was discovered, to 29 January 2021. PE as defined by the trialist was considered sufficient for inclusion. All papers dealing with other forms of hypertension during pregnancy other than PE, including gestational hypertension, chronic hypertension, and chronic hypertension with superimposed PE were excluded. Leptin from any origin (maternal, placental, fetal or neonatal <28 days after birth) was sufficient for inclusion. Both singleton and multiple pregnancies were included. Pre-eclamptic pregnant women of all weight classes were included. All primary studies, regardless of publication type, as well as all types of systematic reviews were included. The search was conducted over a one-month period in February 2021. Titles and abstracts of citations were initially screened. Subsequently, all articles considered relevant underwent full-text evaluation. Finally, a manual search of reference lists of included full-text and review studies was carried out to identify additional relevant articles. This review should be considered a narrative rather than a systematic review.

## 2. Pre-Eclampsia—A Severe Complication of Pregnancy

PE is a serious pregnancy complication and a leading cause of maternal and fetal morbidity and mortality with a prevalence of 3–5% in the western world [7]. In places with limited access to healthcare, e.g., in large parts of Asia, Africa, and South America, between one-tenth and one-quarter of maternal deaths are associated with hypertensive disorders of pregnancy [8]. The background for PE is both complicated and heterogenous [7]. Aberrations in multiple interlocking systems result in acute multisystem maternal disease and compromised fetal growth and development [7]. There is also clear evidence that PE predisposes both mothers and offspring to an array of deleterious health outcomes later in life, including cardiovascular disease [9,10], metabolic syndrome, and obesity [11].

PE is diagnosed with the presence of high blood pressure (systolic > 140 mmHg, diastolic > 90 mmHg) after gestational week 20 in combination with proteinuria (>300 mg/day) or other maternal organ dysfunction, such as impaired liver and kidney function, neurological complications, hematological disturbances, uteroplacental dysfunction, and/or FGR [12]. A more serious presentation of the PE spectrum is the HELLP syndrome, defined by the presence of hemolysis, elevated liver enzymes, thrombocytopenia and/or eclampsia [12]. Some of the established risk factors are increased pre-pregnancy body mass index (BMI), chronic hypertension, pre-gestational diabetes mellitus, previous PE, nulliparity, multi-fetal pregnancy, maternal age >40 years, inter-pregnancy interval >5 years, and assisted reproduction [13].

The pathophysiological background of PE is not fully understood, but the presence of the placenta is necessary and symptoms resolve after delivery [7,14]. Even though the clinical features of PE first become apparent in the second half of pregnancy, major pathogenic changes occur early on at the time of trophoblast invasion and remodeling of the spiral arteries [15]. Multiple factors associated with the establishment of the placenta and essential in fetal-maternal interactions are subsequently compromised [7,14]. These converge to activate and perpetuate uterine-placental dysangiogenesis, immunological imbalances, metabolic disturbances, oxidative stress, systemic endothelial cell activation, and an exaggerated inflammatory response [7,14,16]. Placentas from pre-eclamptic women show the visual signs of the injuries incurred [17].

## 3. Fetal Growth Restriction in Pre-Eclampsia

FGR is defined as the failure of a fetus to reach its genetically determined growth potential [18] and is diagnosed using gestational age-corrected population-based standard growth curves [19]. For this review, FGR is confined to those infants who experience intrauterine growth restriction due to defective fetal-maternal interactions and not those who are constitutionally small-for-gestational-age (SGA) or with fetal genetic or congenital abnormalities. In this sense, FGR is often a consequence of placenta insufficiency, where inadequate transfer of essential substrates, such as oxygen, glucose, amino acids, and fatty acids across the placenta, affects fetal development and growth [20]. The functional capacity of the placenta is dependent on multiple factors, including placenta size and morphology, uteroplacental blood supply, growth factors, and other hormonal and molecular processes that act in nutrient transport over membranes, as well as maternal nutrient availability [20].

Unlike normotensive pregnancies, where roughly 8–14% are affected by FGR [21], up to one-third of pre-eclamptic pregnancies are associated with FGR [15]. The more common late-onset PE (i.e., ≥gestational week 34) typically exhibits normal placental morphology and is less associated with FGR and altered umbilical blood flow [22,23]. The less common early-onset PE (i.e., <gestational week 34) resembles more a placental disease in that placenta morphology is altered [23], umbilical blood flow is insufficient, and FGR is more often apparent [22]. In the same way, some women develop FGR without maternal pathophysiological complications, while others manifest with both PE and FGR [16,24]. The central role of the placenta is undisputed in all cases [16,17], but such differences suggest that different pathologies within shared pathways might be involved [16,21,22,23,24,25]. It has been suggested that PE may result from maternal metabolic syndrome, with aspects of adiposity, insulin resistance, hyperglycemia, leptin resistance, hyperlipidemia, and coagulopathy being of importance. FGR, on the other hand, is suggested to develop in the absence of antenatal metabolic syndrome [24].

## 4. An Introduction to Leptin and Its Receptors

Leptin (from the Greek word leptos, meaning thin) was successfully cloned in 1994 [26]. It is the protein product of the obesity (Lep^ob^) gene, located on chromosome 7, which transcribes a 167 amino acid polypeptide with a molecular weight of 16 kDa. Leptin is synthesized predominantly by adipocytes in white adipose tissue into the circulation and positively reflects adipose tissue size [27]. The classical role of leptin is to provide information about energy stores via its receptors in the hypothalamus and thereby reduce appetite and food intake if energy stores are full. Fasting decreases circulating leptin levels, while feeding or obesity increases leptin levels [27]. Leptin also plays a vital role in the maintenance of normal energy homeostasis by increasing energy expenditure, thus reducing body fat stores [27].

Both the expression of leptin receptors and the production of leptin occurs in many tissues—centrally in the hypothalamus and peripherally in tissues including endometrium, placenta, umbilical cord, endothelium, kidney, muscle, liver, and bone [28]. Leptin acts within a wide range of local and systemic systems with roles within lipid metabolism, insulin sensitivity, angiogenesis, vascular function and blood pressure regulation, bone and cartilage growth, immunity, inflammatory response, puberty onset, fertility, reproductive function, placenta development, pregnancy, and in the intrauterine development of the fetus [2,28,29,30]. Leptin expression and secretion are regulated by many factors, e.g., inflammatory cytokines, glucocorticoids, insulin, norepinephrine release, and beta-adrenergic receptor activation and depend on the leptin source of production and function [27].

The leptin receptor is a member of the class I cytokine receptor superfamily [27]. Six isoforms of leptin receptor have been described and are divided into three classes—long, short and secretory—depending on structural differences [27]. Only the long isoform has full signaling capabilities and is able to activate the Janus tyrosine kinase (JAK/STAT) pathway, the major pathway used by leptin to exert its effects [31]. However, some signaling events can be initiated by the short isoforms. Besides JAK/STAT, other pathways, such as mitogen-activated proteinkinase (MAPK) and the 5′-AMP-activated protein kinase (AMPK) pathway, are also involved in leptin signaling [27,32].

## 5. Leptin and Fetal Growth in Normal Pregnancy

Leptin produced by placental tissues and by maternal and fetal adipocytes plays an integral role in fetal growth in normal pregnancy [2,3,4,5]. Leptin is crucial in placental development and function; it regulates the formation of the blastocyst and has a critical role in implantation and placentation, where it induces human chorionic gonadotrophin production in trophoblast cells and modulates proliferation, protein synthesis, invasion, and apoptosis in placental cells [2,5,6].

Pregnancy is a state of hyperleptinemia in the mother and is closely connected to maternal insulin resistance and nutrient mobilization from maternal fat stores in order to meet the energy demands of the growing fetus [33]. Leptin also acts in placental nutrient transfer by stimulating amino acid uptake [34]. Similar to adults, leptin acts as a signal of fetal energy stores to the fetal central nervous system (CNS) [35]. Furthermore, a variety of mainly animal studies have shown that leptin is an essential growth factor in the development of the CNS, cardiovascular system, renal system, and endocrine system [36].

### 5.1. Maternal Leptin

Maternal serum leptin concentrations are naturally upregulated in pregnant women [33]. Serum leptin concentration is two- to three-fold higher in pregnant women compared to nonpregnant counterparts and peaks at around 28–32 weeks of gestation [37]. Immediately after delivery, levels rapidly decrease back to pre-gestational levels [37]. The significant increase in maternal circulating leptin during pregnancy is caused by increased leptin production from adipose tissue in connection with weight gain and fat deposition in the mother from late second trimester and onwards as well as by placental leptin production already in the first trimester and constitutes about 15% of the total maternal serum leptin concentration [38].

### 5.2. Fetal Leptin

Circulating fetal serum leptin is derived from fetal adipose tissue and from the placenta [35,38]. Leptin has been detected in fetal serum as early as 18 weeks of gestation [39] as well as in fetal adipose tissue biopsies at 20 weeks of gestation [35]. The development of adipose tissue and the accumulation of fat mass are the major determinants of fetal and neonatal serum leptin levels and, as in adults, cord blood leptin level can be taken as a marker of fat mass in the newborn [35,40]. Fetal fat deposition in the third trimester is accompanied by a dramatic increase in fetal serum leptin level [39,41]. Leptin levels are thus positively correlated with birth weight and gestational age [35,42]. In addition, sex is also a major determinant of fetal circulating leptin levels where female fetuses have been found to have higher leptin concentrations than male fetuses [43]. This difference may be the result of a steroid effect, where testosterone in male fetuses suppresses leptin production and release from adipocytes [44]. On the other hand, the limited variation in androgen levels between the sexes at birth [45] suggests that other factors may be responsible for the higher cord blood leptin levels seen in female newborns.

The finding of higher leptin concentrations in umbilical vein blood compared to umbilical arteries [46] and a decline in neonatal serum leptin concentration immediately after delivery [35,46] suggest that the placenta is a source of leptin in the fetal circulation. However, perfusion studies reveal that the placenta makes only a small contribution to the fetal circulation [35,38]. Further, cord blood leptin concentrations are significantly lower than maternal leptin concentrations [43]. It is generally accepted that no correlation between maternal and fetal leptin concentrations exists [47]. Furthermore, maternal leptin levels may or may not be related to fetal birth weight and fat mass in normal pregnancies [48,49]. In cases with increased pre-pregnancy weight and excessive maternal weight gain during pregnancy, however, an increased risk for cord hyperleptinemia has been documented [42] and challenges the hypothesis of a noncommunicating, two-compartment model of leptin regulation across the placenta [47].

### 5.3. Placental Leptin

The placenta is the second largest leptin-producing tissue after adipose tissue in humans and expresses leptin from seven weeks after fertilization [50]. BeWo cells, a choriocarcinoma cell line, and trophoblasts maturing into syncytiotrophoblasts have been identified as a leptin-producing cells [1]. Leptin is also secreted by human amnion cells (amniocytes and decidua) into amniotic fluid [1]. As well as being a source of leptin, the placenta is also a target of leptin action [6]. The human placenta expresses a high amount of leptin receptor mRNA from early gestation, i.e., gestational weeks 7–14, up to term [38,51]. The presence of both leptin and leptin receptors in the placenta suggests that leptin has an auto/paracrine role in the human placenta [51].

Placental leptin production increases with gestational age [52] and contributes to maternal and fetal circulating levels during pregnancy [35,38]. According to placental perfusion studies, 95–98.4% of placental leptin is released into the maternal circulation while only 5.0% or less enters the fetal circulation [35,38]. Due to the molecular weight of leptin, maternal leptin cannot cross over the placental barrier freely and thus does not contribute to leptin levels in the placenta, amniotic fluid or fetus. However, the location of transmembrane leptin-receptors on placental cells facing the maternal circulation implies that receptors are likely to bind maternal circulating leptin [51].

## 6. Leptin in Pre-Eclampsia

Leptin is unquestionably involved in the pathophysiology of PE [2,3,4,5,53,54]. The more detailed evidence of leptin’s functions in PE is, however, often conflicting or lacking. Contradictory evidence may be due to differing criteria for the diagnosis of PE, differing time points in pregnancy when leptin was measured, small sample sizes, lack of differentiation between early- and late-onset PE, differing degrees of PE severity, varying measurement techniques, lack of correction for important confounders such as maternal BMI, gestational age-corrected birth weight/pondural index, prenatal administration of steroids, and variation in participant characteristics such as smoking and ethnicity. Furthermore, the possibility that homoeostatic mechanisms become unbalanced outside, e.g., the normal maternal and/or fetal weight range, can explain why studies vary so much in their maternal-fetal leptin correlation findings.

### 6.1. Maternal Leptin in PE

The large majority of studies on serum leptin concentrations in women with clinical PE have found upregulated levels in the late second and third trimester [55,56,57,58,59,60,61,62]. Compared to pregnant women with uncomplicated pregnancies, in PE leptin does not peak in week 28–32, but continues to increase markedly, only falling again after delivery [37]. Increased leptin levels in the maternal circulation may originate from increased production in the placenta, but there are no existing placental perfusion studies to confirm this. Other studies have, however, found lower [63,64,65] or similar [66,67,68] maternal serum leptin levels in women with clinical PE compared to normotensive counterparts. Perturbed maternal leptin levels before symptom onset have also been found, with some studies reporting elevated leptin levels [37,69,70,71] and others reduced leptin levels in the first and early second trimester [72]. Others again have found no significant alternation in maternal serum leptin level prior to clinical symptoms [73].

Maternal serum leptin concentrations may or may not correlate negatively with birth weight in pre-eclamptic pregnancies [56,57,62,65,71]. Further, conflicting findings exist regarding whether there is a positive correlation between maternal and fetal serum leptin concentrations, but it seems unlikely [65,74]. The elevation of maternal serum leptin is most likely proportional to the severity of PE. Several studies found that maternal serum leptin levels were increased in women with severe PE [55,59,60,75]. However, others reported the opposite [76] or no association between leptin level and severity [67,77]. Furthermore, differences in disease severity due to ethnic differences could not be explained by differences in leptin concentration [78]. Finally, the leptin profile may be different in early- and late-onset PE [79], with higher levels found in women with early-onset PE in some studies [58,60]. This is in contrast to other studies with findings of higher maternal serum leptin levels in term PE compared to pre-term PE [56,69].

### 6.2. Fetal Leptin in PE

Theoretically, one might expect to find lower levels of cord blood leptin in infants of pre-eclamptic mothers compared to infants of normotensive mothers, especially in cases complicated by FGR, where infant body fat mass and body weight are reduced. On the other hand, placental leptin levels are increased in PE and may contribute to the fetal circulation and result in excessively high fetal serum leptin concentrations. Again, evidence is conflicting. Some studies revealed no difference in cord blood leptin concentration in infants of pre-eclamptic and normotensive mothers [65,74,80], including those with FGR [81]. Others reported lower cord blood leptin levels in infants of pre-eclamptic women, which suggests that placental leptin does not make a major contribution to fetal levels [35,63,64,68]. The largest study to date, with 256 PE cases and 607 controls, found higher levels of cord blood leptin in infants of mothers with PE compared with infants of control subjects [82].

Similar to normotensive pregnancies, the cord blood leptin concentration in infants of pre-eclamptic mothers has been found to correlate positively with birth weight, pondural index, and female sex [65,80,82]. At present, there seems to be no clear difference in cord blood leptin levels and varying severity of PE [82].

### 6.3. Placental Leptin in PE

There are significant increases in leptin mRNA expression and leptin protein in the placentas of pre-eclamptic mothers [56,75,83]. Compared to normotensive controls, placental leptin production is increased eightfold [81]. Furthermore, leptin mRNA and protein in pre-eclamptic placentas seem to be higher in preterm births than in term births [56]. Leptin gene expression is differentially upregulated by 40-fold in severe pre-eclamptic placentas compared to normal placentas in both early- and late-onset PE and pathways involving oxidative stress and inflammation, and endothelin, cytokine, and chemokine signaling have been identified as possible contributing mechanisms [84]. Placental leptin gene expression has been found to correlate negatively with birth weight, but no correlation between placental leptin level and birth weight has been found [56].

## 7. Leptin and Fetal Growth in Pre-Eclampsia

**Figure 1:**
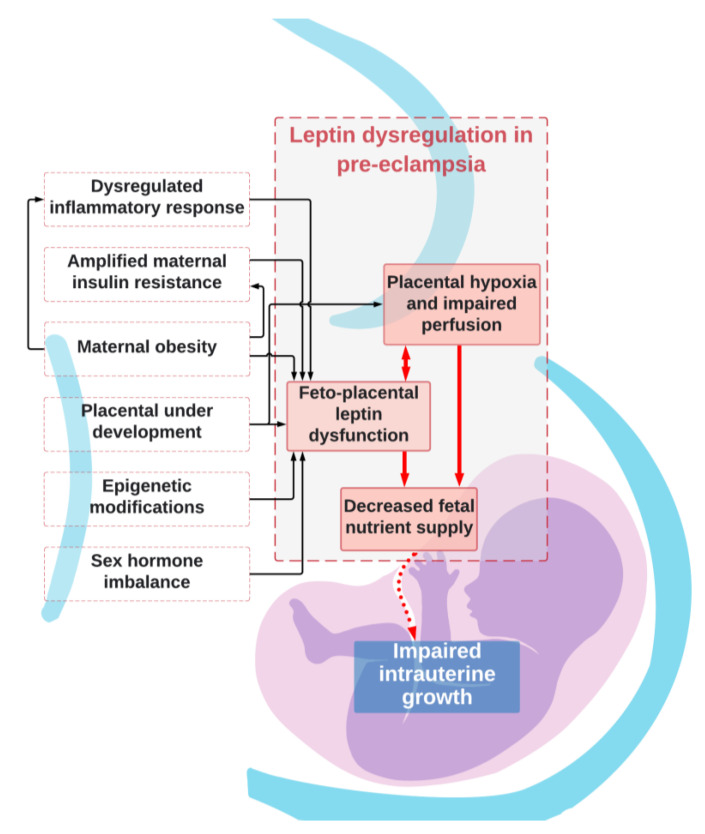
Leptin dysregulation in pre-eclampsia.

Leptin is dysregulated in both PE and FGR [2,3] and is influenced by a number of factors (Figure 1). Dysregulated leptin production and/or its receptors may also be connected to FGR in pregnancies complicated by PE. The identification of any possible unifying pathologic mechanisms of leptin in these conditions is, however, challenging. Firstly, even though PE is frequently related to FGR, not all pre-eclamptic pregnancies result in growth restricted neonates, just as not all pregnancies with FGR result in PE in the mother. We have yet to fully understand this dichotomy. Secondly, much of the evidence regarding leptin in FGR and leptin in PE is conflicting. Thirdly, there are currently no sufficient data regarding the role of leptin in fetal growth in pre-eclamptic pregnancies with and without FGR [71].

### 7.1. Maternal Leptin and Fetal Growth in PE

Studies have reported both lower [85] and higher [57,73,86] maternal leptin levels in normotensive women with growth-restricted neonates compared to normotensive women with appropriate-for-gestational-age (AGA) neonates. Other studies of normotensive women found no correlation between maternal leptin level and fetal weight [56,78,81]. Contrarily, a negative correlation between maternal serum leptin levels and ∆SD of body weight of neonates has been reported, suggesting that maternal serum leptin reflects the severity of SGA in association with placental dysfunction in normotensive pregnant women [57]. In pre-eclamptic women with pregnancies complicated by FGR, maternal serum leptin levels were reported as higher in cases with FGR compared to cases without FGR [57,73,87]. Further, the incidence of FGR in pre-eclamptic women was higher if serum leptin levels were high (>+1.5 SD) than in women with normal leptin levels [87]. The increased serum leptin of pre-eclamptic women could reflect the overproduction of leptin from a placenta suffering from placental dysfunction [81,86], which in itself cannot sustain normal fetal growth, resulting in FGR.

### 7.2. Fetal Leptin and Fetal Growth in PE

Lower cord blood leptin levels have been reported in growth restricted neonates of normotensive women compared to healthy controls [39,41,85]. Reduced fetal fat mass as well as a possible lower contribution of leptin from a dysfunctional placenta may explain these findings. The decreased leptin levels in cord blood could also be a compensatory mechanism against placental overproduction [81,86] and it has been suggested that the binding capacity of leptin in the newborn is high due to the upregulation of leptin binding receptors in response to high placental leptin production [88]. All the above hypotheses may also explain the mechanisms of FGR in PE, but sufficient data do not exist specifically addressing fetal growth in PE. Little is known about how intrauterine stressors such as PE affect the expression of fetal leptin and its receptors or how fetal fat accretion is regulated. However, we do know that fetal leptin is a marker of fetal fat mass in AGA, SGA, and large-for-gestational-age infants [35,39,40]. Thus, fetal leptin levels are most likely a consequence rather than a stimulus of fetal growth, and the role of leptin as a growth hormone is probably limited to specific fetal tissues rather than general growth [4].

### 7.3. Placental Leptin and Fetal Growth in PE

In contrast to PE, both decreased [89] and similar [56] leptin mRNA expression and leptin protein in placentas from normotensive women with SGA neonates have been reported compared to healthy controls. Increased placental leptin expression and protein levels have also been reported in normotensive women with SGA neonates, but these were still significantly lower than in pregnancies complicated by severe PE (mRNA leptin expression two times higher and placental leptin level three times higher in severe pre-eclamptic pregnancies compared to normotensive pregnancies associated with FGR) [81]. It would appear that, despite the common pathological background of defective placentation and deficient spiral artery remodeling in both FGR and PE, other mechanisms are involved and no common pattern in placental leptin expression or production is evident as of yet. There are insufficient data available regarding placental leptin in PE associated with FGR, but a study of leptin in amniotic fluid has been conducted [90]. Here, increased amniotic fluid concentrations of leptin were found in women who later developed PE, and leptin concentrations were significantly higher in pre-eclamptic pregnancies associated with FGR. In normotensive pregnancies, there was no association between amniotic fluid leptin level and fetal growth, and levels were dictated by maternal BMI [91].

## 8. Mechanisms for Dysregulated Leptin in Pre-Eclampsia and Effects on Fetal Growth

**Figure 2:**
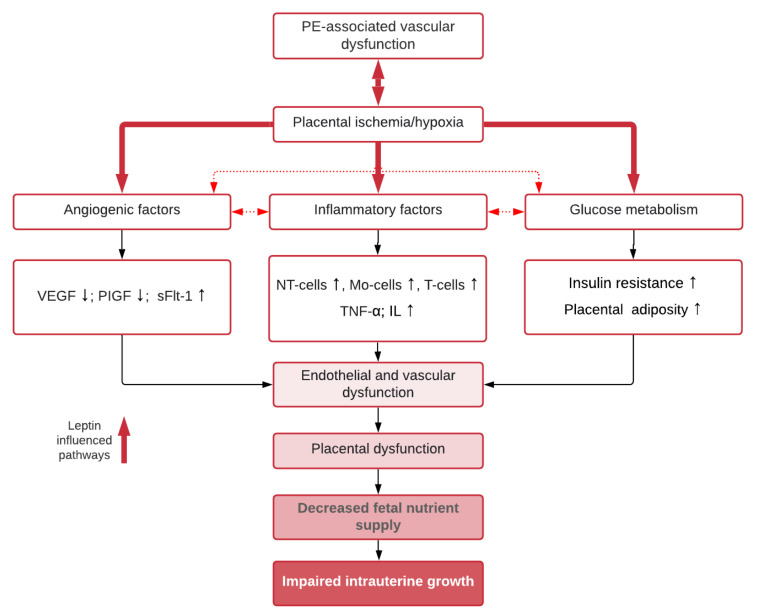
Key leptin-regulated pathways in impaired intrauterine growth in pre-eclampsia. Abbreviations: VEGF: Vascular Endothelial Growth Factor; PIGF: Placental Growth Factor; sFlt1: Soluble Fms-like Tyrosine Kinase-1; NT-cells: Neutrocytes; Mo-cells: Monocytes; TNF-α: Tumor Necrosis Factor alpha; IL: Interleukins.

**Figure 3:**
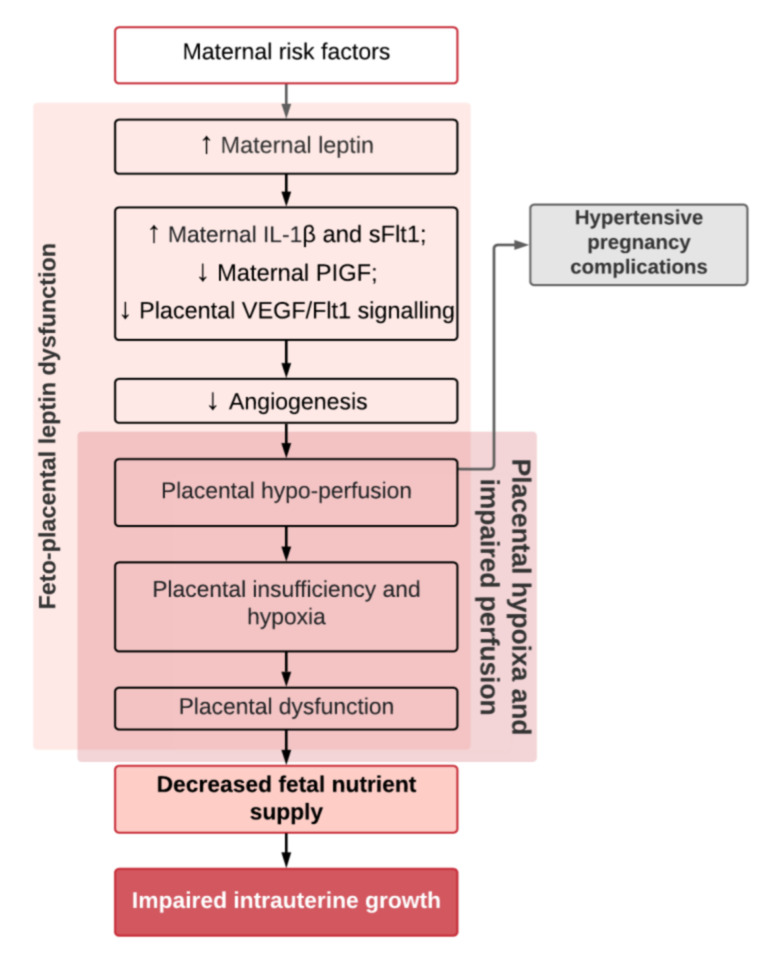
Leptin and dysangiogenesis in pre-eclampsia. Abbreviations: IL-1β: Interleukin-1 beta; sFlt1: Soluble Fms-like Tyrosine Kinase-1; PIGF: Placental Growth Factor; VEGF/Flt1: Vascular Endothelial Growth Factor/Fms-like Tyrosine Kinase-1.

The exact mechanisms involving leptin in the pathophysiology of PE remain elusive and there is uncertainty about whether leptin represents the complications of the established PE or if leptin dysregulation is a catalyst for disease onset. Regardless, there are multiple potential stages in pregnancy where dysregulated leptin levels may impact fetal growth (Figure 1):

1. Leptin has auto- and paracrine roles in placental development and function where it regulates implantation and placentation and modulates functions in placental cells [4,51]. Abnormal trophoblast proliferation or invasion has been associated with abnormal placental leptin release in pregnancies complicated by FGR [89] and may also be involved in placental growth in PE.
2. Leptin upregulates amino acid transport activity across the placenta [34]. Leptin is also known to stimulate lipolysis in the placenta, which may impact free fatty acid availability to the fetus [92]. Dysregulation of placental leptin in PE may thus affect nutrient supply and fetal growth.
3. It has been suggested that placental production of leptin is augmented in PE because of placental hypoxia, which is a consequence of reduced placental perfusion (Figure 2) [75]. Hypoxia has been proven to be a positive regulator of placental leptin gene expression and production [75]. Placental hypoxia-inducible factor 1alpha (HIF1alpha) mRNA level and level of placental leptin mRNA expression are positively correlated [93], which implies that the rapid increase in maternal serum leptin levels seen in the third trimester of pre-eclamptic pregnancies [37] may be, at least in part, explained by placental hypoxia. Alternatively, abnormalities within HIF1alpha’s oxygen sensing properties, rather than hypoxia, may promote conditions for the development of PE [94]. Either way, it remains unclear what impact this has on fetal growth, as pregnancies complicated by FGR have not typically been characterized by high placental leptin mRNA expression and leptin protein levels [56], which suggests that pregnancies complicated by FGR do not suffer from placental hypoxia as in PE.

4. Exaggerated maternal hyperleptinemia in PE may be a compensatory response to impaired placental perfusion in order to boost nutrient delivery to the fetus [56] and is supported by findings of an increased placental leptin content and a positive correlation with the resistance index of the umbilical artery in both PE and FGR [81]. Furthermore, animal experiments with rats showed significantly increased maternal serum leptin levels in animals with reduced placental perfusion [95]. It has been hypothesized that PE occurs in women who cannot accommodate for or tolerate the exaggerated increase in leptin whilst FGR occurs in women who do not respond enough to compensatory hyperleptinemia [56].
5. Leptin is both associated with angiogenic molecules in PE [61] and is itself a angiogenic factor that can enhance vascular endothelial growth factor synthesis during pregnancy (Figure 3) [96]. Maternal serum leptin and endothelin-1 (a highly potent vasoconstrictor that is released in conditions of hypoxia) were found to be increased in women with PE and were correlated positively with the degree of FGR in women with PE [97]. The increase in leptin may be a response to under-perfusion of the placenta in an attempt to support neovascularization and improvement of nutrient delivery [2,4,61,81].

6. Increased insulin resistance from the second trimester and onwards ensures adequate nutrient supply to the growing fetus and is regulated by a range of maternal, placental, and fetal hormones [33]. However, in pregnancies complicated by PE, insulin resistance is amplified above the normal range [98]. Insulin is a positive regulator of leptin production [99], and it has been speculated that maternal hyperinsulinemia upregulates placental leptin gene expression and production in pre-eclamptic women (Figure 2) [56,100]. Furthermore, the development of insulin resistance can originate from adipose tissue in pre-eclamptic women with overweight or obesity [101]. Fetal growth may be affected by perturbed maternal energy balance and reduced glucose availability resulting from increased leptin and insulin resistance [87]. Metabolic disturbances involving leptin and insulin may also trigger a cascade of changes within compensatory mechanisms in immune, inflammatory, and endothelial pathways that ultimately end in placental insufficiency and FGR [102].
7. Overweight and obesity are known risk factors for PE [55,103]. Several studies have proposed that the normal relationship between serum leptin concentrations and adiposity is disrupted in PE and is potentiated by increasing BMI [62,69,72,104]. As in lean women, PE in maternal obesity is associated with FGR rather than fetal overgrowth, which otherwise is common in normotensive pregnancies [105]. There are several hypotheses as to how leptin might be involved in the increased risk of PE and FGR in overweight and obese women. In pregnant women with BMI values on the extreme ends of the scale, normal compensatory mechanisms may be inadequate in controlling metabolic homeostasis [72]. Increased insulin resistance originating from adipose tissue can be a contributing factor [101]. Placental insulin resistance may reduce amino acid transfer across the placenta [104]. Finally, obesity is itself an inflammatory condition where increased production of leptin, proinflammatory cytokines, and complement proteins from adipose tissue can aggravate endothelial dysfunction and cause reductions in placental perfusion [106].
8. An exaggerated maternal systemic inflammatory response to pregnancy with activation of both the innate and the adaptive immune system plays a central role in the pathogenesis of PE [107]. The increased shedding of syncytiotrophoblast microparticles from the placenta, persistent hypoxic conditions, and/or alterations in oxygen-sensing mechanisms in the placenta may promote activation of maternal leukocytes and endothelial cell dysfunction [108]. Class 1 cytokines, which induce inflammation, are upregulated and class 2 cytokines, which regulate inflammation, are down-regulated in PE [107]. Leptin has also been shown to play a role in immunity [109]. Inflammatory stimuli or immune dysfunction could alter maternal leptin expression in PE (Figure 2) [110]. Leptin is itself a class 1 cytokine with proinflammatory properties. There is evidence that inflammatory mediators increase serum leptin concentrations [111] and that leptin contributes to the increased circulating levels of pro-inflammatory mediators in PE [2,112].
9. Epigenetic modifications with differential placental and fetal gene expression for leptin and its receptors, as well as factors involved in cytokine and endothelin signaling pathways, protein modification, and regulation of JAK-STAT have been reported for both PE and FGR [6,84]. Furthermore, transmembrane leptin receptors in syncytiotrophoblast and trophoblast cells are accessible to maternal leptin (of both placental and maternal adipose origin) and may signal differently in pregnancies with increased leptin production [51]. Further, the increased expression of a soluble leptin receptor in cytotrophoblast cells of pre-eclamptic placentas may modify leptin-binding capabilities and may modulate free leptin levels [51]. Further examination of genetic variations in leptin and its receptors is warranted to understand the possible effect on fetal growth.
10. Finally, steroid hormones are a positive regulator of placental leptin production [54]. The sex-hormone imbalance found in PE pregnancies with elevated estradiol might lead to an increase in the maternal serum levels of leptin in PE [113].

## 9. Conclusions

This review examines leptin dysregulation in PE within maternal, fetal, and placental compartments and presents possible mechanisms of leptin action in the complex web of interactions that characterizes PE. This review illustrates that dysregulated leptin in all three compartments is linked to aberrant intrauterine growth in PE. Metabolic disorders and insufficient homeostatic compensatory mechanisms involving leptin during pregnancy appear to play a decisive role in the development of compromised intrauterine growth conditions and link leptin to multiple parallel systems. There is not, however, any single set of unifying mechanisms involving leptin regulation/dysregulation that can explain aberrant intrauterine growth within the spectrum of pre-eclamptic disorders or in FGR outside of PE. These pathologic conditions are heterogenous, and modifications in regulatory mechanisms involving leptin are specific to each situation. Nevertheless, leptin is involved in fetal growth at many levels and probably throughout the entirety of pregnancy in all cases.

More studies that examine fetal growth in PE are needed. Furthermore, we need to gain a better understanding of leptin action in independent FGR in order to understand how this pathology differs from FGR in PE. The pleiotropic role of leptin and its relationship to factors in metabolic, angiogenic, immune, inflammatory, and endothelial systems in PE need further attention in order to understand fetal-maternal metabolism and fetal-maternal communication across the placenta.
